# Glycoprotein 90K Promotes E-Cadherin Degradation in a Cell Density-Dependent Manner via Dissociation of E-Cadherin–p120-Catenin Complex

**DOI:** 10.3390/ijms18122601

**Published:** 2017-12-02

**Authors:** So-Yeon Park, Somy Yoon, Eun Gene Sun, Rui Zhou, Jeong A. Bae, Young-Woo Seo, Jung-Il Chae, Man-Jeong Paik, Hyung-Ho Ha, Hangun Kim, Kyung Keun Kim

**Affiliations:** 1 College of Pharmacy, Sunchon National University, 255 Jungang-ro, Sunchon, Jeonnam 57922, Korea; sinbu17@naver.com (S.-Y.P.); zhourui274@hotmail.com (R.Z.); paik815@sunchon.ac.kr (M.-J.P.); hhha@sunchon.ac.kr (H.-H.H.); 2 Medical Research Center for Gene Regulation, Brain Korea 21 Project, Chonnam National University Medical School, 160 Baekseo-ro, Dong-gu, Gwangju 61469, Korea; oouate@naver.com (S.Y.); cannon0204@hanmail.net (E.G.S.); risstuu@empal.com (J.A B.); 3 Korea Basic Science Institute, Gwangju Center, 77 Yongbong-ro, Buk-gu, Gwangju 61186, Korea; whitefox@kbsi.re.kr; 4 Department of Dental Pharmacology, School of Dentistry and Institute of Oral Bioscience, BK21 Plus, Chonbuk National University, 567 Baekje-daero, Jeonju, Jeonbuk 54896, Korea; jichae@jbnu.ac.kr

**Keywords:** 90K glycoprotein, LGALS3BP, Mac-2BP, E-cadherin, p120-catenin, adherens junction

## Abstract

Glycoprotein 90K (also known as LGALS3BP or Mac-2BP) is a tumor-associated protein, and high 90K levels are associated with poor prognosis in some cancers. To clarify the role of 90K as an indicator for poor prognosis and metastasis in epithelial cancers, the present study investigated the effect of 90K on an adherens junctional protein, E-cadherin, which is frequently absent or downregulated in human epithelial cancers. Treatment of certain cancer cells with 90K significantly reduced E-cadherin levels in a cell-population-dependent manner, and these cells showed decreases in cell adhesion and increases in invasive cell motility. Mechanistically, 90K-induced E-cadherin downregulation occurred via ubiquitination-mediated proteasomal degradation. 90K interacted with the E-cadherin–p120-catenin complex and induced its dissociation, altering the phosphorylation status of p120-catenin, whereas it did not associate with β-catenin. In subconfluent cells, 90K decreased membrane-localized p120-catenin and the membrane fraction of the p120-catenin. Particularly, 90K-induced E-cadherin downregulation was diminished in p120-catenin knocked-down cells. Taken together, 90K upregulation promotes the dissociation of the E-cadherin–p120-catenin complex, leading to E-cadherin proteasomal degradation, and thereby destabilizing adherens junctions in less confluent tumor cells. Our results provide a potential mechanism to explain the poor prognosis of cancer patients with high serum 90K levels.

## 1. Introduction

The protein known as lectin galactoside-binding soluble 3-binding protein (LGALS3BP), Mac-2-binding protein (Mac-2BP), or 90K is a secretory glycoprotein, and its serum levels are observed to be elevated in several cancers and viral infections. As 90K plays a role in immune defense and immune regulation, its levels are therefore increased in patients with human immunodeficiency virus [1], hepatitis C virus [2], hantavirus [3], or dengue virus [4] infection. 90K was originally identified as a tumor-associated antigen that is released in the culture media of human breast cancer cells [5], and it has both negative and positive effects on the prognosis of various cancers [6]. The upregulation of 90K in several cancers including colon, prostate, gastric, lung, breast, liver, pancreas, and oral cancers suggests an association between 90K and tumorigenesis [7,8,9,10,11,12,13,14,15]. A high (>11 µg/mL) serum level of 90K in breast cancer patients was reported as a poor prognostic factor [16] and associated with distant recurrence [12], and enriched 90K was found from secreted exosomes of ovarian carcinoma cells [17]. Increased expression of 90K by immunohistochemistry is correlated with poor disease-free and overall survival in patients with non-small-cell lung cancer [11]. On the other hand, a suppressive effect of 90K on tumor progression is evident in some reports. For example, the lack of 90K expression is associated with disease aggressiveness and shorter survival in laryngeal cancer [6] or colorectal cancer [18]. 90K is downregulated in advanced colorectal cancer (CRC) tissues and invading cancer cells of corresponding metastatic liver tissues [19]. Taken together, these findings indicate that the role of 90K in cancer is dependent on the specific microenvironment of differentiated cancer cells.

Regarding the mechanisms underlying the effect of 90K on tumor progression, it was reported that 90K activates early immune responses to tumors and suppresses Wnt signaling via CD9/CD82-mediated ISGylation-dependent ubiquitination of β-catenin in CRC cells [19,20]. However, the antitumor activity of 90K is neutralized in advanced CRC cells and tissues through interaction with galectins, which are highly expressed in tumor cells. Circulating 90K may have a tumor metastasis-promoting effect by acting as a glue to increase the aggregation of tumor cells expressing high levels of galectins [6]. However, the interaction of galectins with 90K is not sufficient to explain the role of high serum 90K levels as a poor prognostic factor in several cancers; therefore, other oncogenic mechanisms of 90K may be active under certain tumor microenvironments. For example, a recent report demonstrated that 90K, through the interaction with Wnt proteins, may act as a reservoir of Wnt in the extracellular matrix (ECM) of various tissues, and can thereby regulate downstream signaling of the Wnt pathway [21]. Also, 90K was found to trigger integrin signaling by acting as a ligand of integrins, and cellular exposure to 90K significantly increases viability, motility, and migration [22].

E-cadherin is a type I transmembrane protein found at adherens junctions that mediates Ca^2+^ dependent cell–cell adhesion. It is associated with the actin cytoskeleton via cytoplasmic proteins, including α-, β-, and p120-catenin, forming the cadherin/catenin complex that is crucial to adherens junctions [23]. E-cadherin is expressed in normal epithelial cells and suppresses tumorigenicity; however, its expression or cell surface localization is often lost in advanced tumors. The intracellular localization and expression of E-cadherin and β-catenin are heterogeneous in tumor cells and reflect the heterogeneity of tumor cell differentiation [24]. Loss of E-cadherin function promotes invasion, metastasis, and the conversion of epithelial tumor cells into highly migratory and invasive cells, and is a hallmark of epithelial-to-mesenchymal transition [25,26].

In the present study, we aimed to elucidate the mechanism underlying the association of 90K with poor prognosis and metastasis of cancer. For this purpose, we examined the effect of 90K on the levels of E-cadherin, which forms the cadherin/catenin complex that is crucial to the function of adherens junctions, in various cancer cells. Our results showed that 90K destabilizes E-cadherin and affects cell adhesion and invasion in subconfluent cancer cells via dissociation of the E-cadherin–p120-catenin complex, suggesting that 90K drives less confluent tumor cells into the early steps of cancer metastasis. Namely, our results provide a mechanistic explanation of distant recurrence in cancer patients exhibiting high serum 90K levels: 90K promotes the release of cancer cells from tumor tissues through the destabilization of the adherens junction.

## 2. Results

### 2.1. 90K Negatively Regulates E-Cadherin Levels in a Cell-Population-Dependent Manner

**Figure 1:**
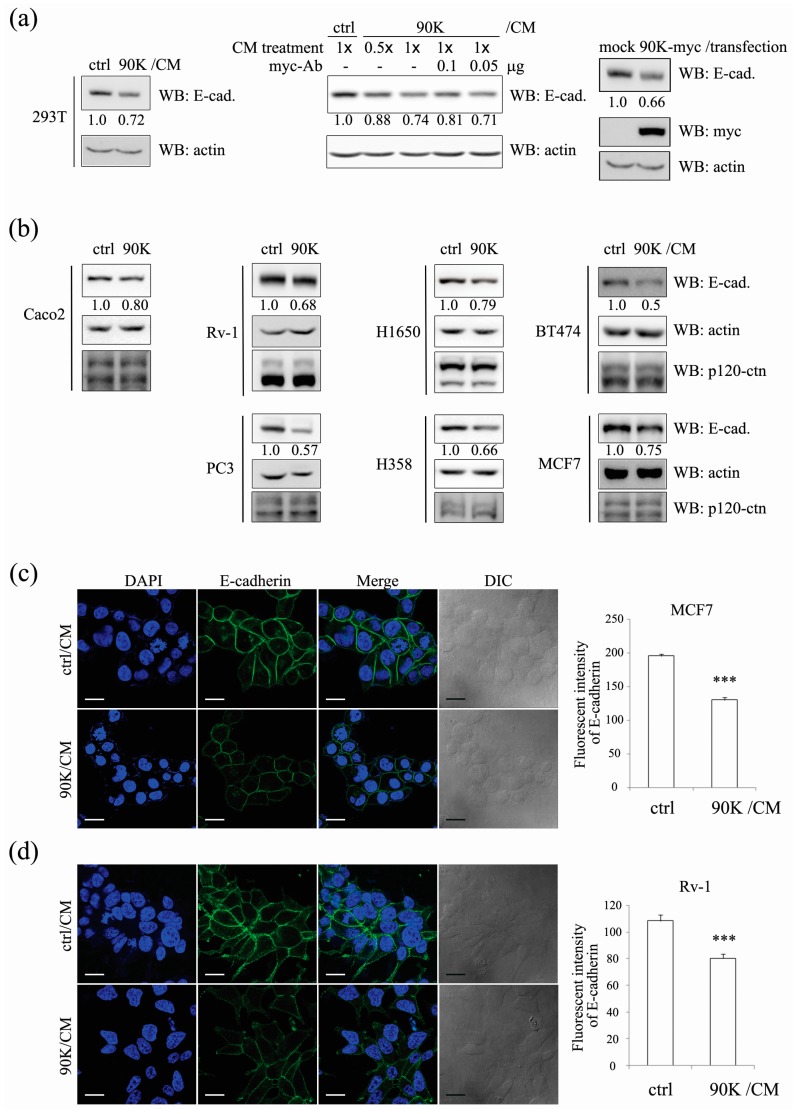
90K negatively regulates E-cadherin levels. (**a**,**b**) 90K conditioned media (90K/CM) treatment or overexpression decreased E-cadherin levels in 293T cells (**a**), Caco2 (colorectal cancer cells), CWR22Rv-1 and PC3 (prostate cancer cells), H1650 and H358 (lung cancer cells), BT474 and MCF7 (breast cancer cells) cells (**b**). For conditioned-media-treated cells, cells were plated to 50% confluence and treated with either control conditioned media (ctrl/CM) or 90K/CM for 16 h. A myc antibody was added to conditioned media to deplete functional 90K-myc from the conditioned media. The “1×” and “0.5×” denote that the cells were treated with a normal or half amount of 90K/CM, respectively (**a**, middle panel). For transfection, cells were plated to 50% confluence and transfected with either mock or 90K-myc plasmid (**a**, right panel). Cell lysates were immunoblotted for E-cadherin, p120-catenin, myc, and actin. Actin was used as a loading control. Relative values of E-cadherin/actin ratio from at least three independent experiments are shown below the E-cadherin panel; (**c**,**d**) Immuno-reactivity for membranous E-cadherin was significantly decreased by 90K/CM treatment in MCF7 (**c**) and CWR22Rv-1 (**d**) cells. Cells were treated with either ctrl/CM or 90K/CM for 16 h and immunostained using an E-cadherin antibody. Representative images are shown in the left panel and corresponding fluorescent intensities of E-cadherin are shown in the right panel. Values are presented as mean ± SEM. An asterisk indicates a significant difference between the 90K/CM and ctrl/CM groups (*** *p* < 0.001). Scale bar, 20 μm.

**Figure 2:**
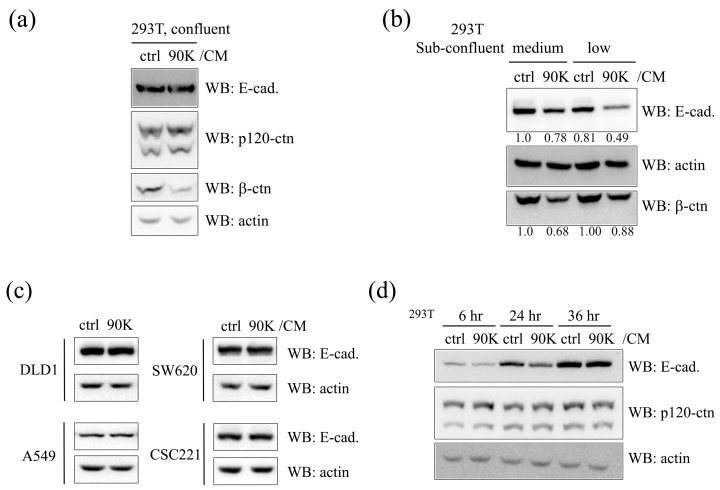
90K decreases E-cadherin levels in a cell-population-dependent manner. (**a**) The effect of 90K on E-cadherin levels was decreased in confluent cells. Cells were plated to 70% confluence; treated with either ctrl/CM or 90K/CM for 16 h; and immunoblotted for E-cadherin, p120-catenin, and β-catenin; (**b**) The effect of 90K on E-cadherin levels was cell-population-dependent. Cells were plated to different confluence levels (medium, 50%; low, 30%); treated with either ctrl/CM or 90K/CM for 16 h; and immunoblotted for E-cadherin, actin, and β-catenin. The effect of 90K on β-catenin levels was also cell-population-dependent, and more evident in confluent cell populations. Actin was used as a loading control. Relative values of E-cadherin/actin and β-catenin/actin ratio in triplicate experiments are shown below the E-cadherin and β-catenin panel, respectively; (**c**) The effect of 90K on E-cadherin levels was decreased in DLD1, SW620, A549, and CSC221 cells. Cells were plated to 50% confluence, treated with either ctrl/CM or 90K/CM for 16 h, and immunoblotted for E-cadherin and actin; (**d**) The effect of 90K on E-cadherin levels was dependent on treatment time. Cells were plated to low confluence; treated with either ctrl/CM or 90K/CM for the indicated times; and immunoblotted for E-cadherin, p120-catenin, and actin.

As the upregulation of 90K in several cancers including colon, prostate, lung, and breast suggests an association between 90K and tumorigenesis [7,9,11,12], and E-cadherin depletion is directly related to migratory and metastatic properties of the cancer cell, here we aimed to analyze the effects of 90K on E-cadherin levels in cancer cell types that are known to exhibit varying degrees of migratory capability. To test the effects of 90K on E-cadherin levels in the above-mentioned cancer types, HEK293T cells, the colorectal cancer cell line Caco2, the prostate cancer cell lines CWR22Rv-1 and PC3, the lung cancer cell lines H1650 and H358, and the breast cancer cell lines BT474 and MCF7 were treated with 90K conditioned medium (90K/CM). As shown in Figure 1a,b, 90K/CM treatment significantly decreased E-cadherin levels in whole cell lysates of several cell lines while the level of p120-catenin remained constant. The effects were dependent on the amount of 90K/CM treatment, and depletion of 90K from the conditioned media by immunolabeling with anti-myc antibody restrained the 90K effect on E-cadherin (Figure 1a, middle panel). Instead of conditioned media treatment, transfection of the 90K expression plasmid to the cell also decreased E-cadherin levels (Figure 1a, right panel). The level of N-cadherin was also decreased by 90K treatment (Figure S1a). It was observed that the levels of membranous E-cadherin were significantly decreased in 90K/CM-treated MCF7 and CWR22Rv-1 cells (Figure 1c,d). Interestingly, however, 90K/CM treatment did not affect E-cadherin levels in confluent cells (Figure 2a), whereas the effects of 90K on E-cadherin levels were particularly evident in low-confluence cells (Figure 2b). This was also the case for N-cadherin (Figure S1b). The opposite effects were observed for β-catenin, namely, 90K decreased β-catenin levels in confluent cells [19,27], whereas its effects were marginal in low-confluence cells (Figure 2a,b). It should be noted that the population of cell or status of cell–cell contact is a greater determining factor for the 90K effect on E-cadherin than the concentration or amount of 90K. Taken together with the diminished effects of 90K on E-cadherin in certain cell lines such as A549, DLD1, SW620, and CSC221 (Figure 2c), these results suggest that the effects of 90K on E-cadherin and, thus, on the formation of adherens junctions depend on the tissue microenvironment. To determine the time course of the effects of 90K on E-cadherin, HEK293T cells were incubated with 90K/CM for different times. The effect of 90K on E-cadherin levels was detected at 6 h, suggesting that the changes may not be transcriptional, whereas it was abolished at 36 h, probably because the cells reached a confluent state (Figure 2d).

### 2.2. 90K Decreases Adhesion and Increases Invasion of Subconfluent Cancer Cells

**Figure 3:**
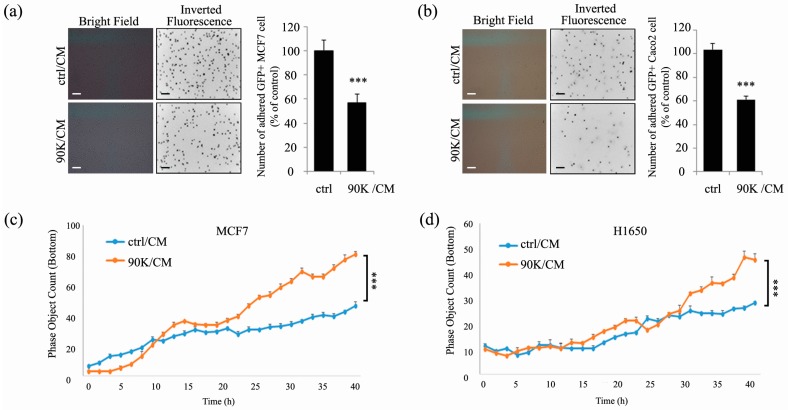
90K decreases adhesion and increases invasion of subconfluent cancer cells. (**a**,**b**) Cell–cell adhesion was decreased by 90K. Cell–cell adhesion was allowed for 1 h with MCF7 (**a**) and Caco2 (**b**) cells. Cells plated on 30% confluence were treated with either ctrl/CM or 90K/CM for 16 h and, then, stained with Calcein green prior to addition into the well where the non-stained confluent cells were plated. A quantity of 3 × 10^5^ stained cells were added into the cell-coated well. Adherent Calcein green-positive cells were photographed by fluorescence microscopy and inverted grayscale images from fluorescence picture are shown. Quantitations of the cell number are shown in the right panel. Scale bar, 100 μm. (**c**,**d**) Invasive potential of the cells was increased by 90K. Invasion assay was performed with subconfluently plated MCF7 (**c**) and H1650 (**d**) cells treated with either ctrl/CM or 90K/CM. Invaded cells at the bottom of transwell chamber were monitored using a live-cell imaging device (IncuCyte) with a real-time check for the subconfluency of the upper chamber cells, and time-course analyses of the invaded cell number are shown. Values are presented as mean ± SEM. *** *p* < 0.001 compared to control group.

As cadherins play important roles in the adhesive and mesenchymal properties of the cells, the functional significance of 90K-induced decreases in cadherin levels on the properties of cancer cells was tested. As shown in Figure 3a,b, 90K/CM treatment to subconfluent cancer cells significantly decreased adhesion between the cells. Also, 90K/CM treatment significantly increased the invasive motility of the cells (Figure 3c,d and Figure S2). During the live-cell imaging analysis of the cell invasion, we noted that the increases in invasive motility of the cell by 90K were observed when cell–cell contact is absent, and, if the cell reaches their confluency, showed rather decreased invasive motility even in the presence of 90K [28]. We also found that 90K/CM treatment significantly decreased adhesion of subconfluent MCF7 and Caco2 cells to various adhesive substrates including fibronectin, collagen, poly-l-lysine, and matrigel matrix (Figure S3). Taken together, these results clearly show the functional significance of the 90K-induced E-cadherin downregulation in low-confluence cancer cells and provide a mechanistic implication of the role of 90K on tumor progression.

### 2.3. 90K Modulates E-Cadherin Levels via Ubiquitination-Mediated Proteasomal Degradation but Not via ISGylation

**Figure 4:**
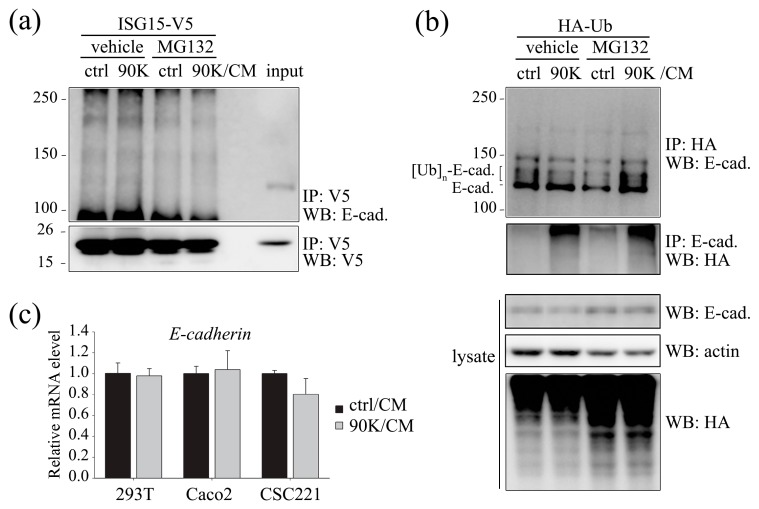
Regulation of E-cadherin level by 90K occurs via ubiquitination-mediated proteasomal degradation and not ISGylation. (**a**) 90K conditioned media (90K/CM) treatment did not induce ISGylation of E-cadherin. 293T cells were transfected with ISG15-V5, treated with either ctrl/CM or 90K/CM for 16 h, and immunoprecipitated with V5 antibody to pull down ISGylated proteins. Bound proteins were subjected to western blot analysis to detect ISG15-conjugated E-cadherin using an anti-E-cadherin antibody; (**b**) 90K/CM treatment promoted E-cadherin ubiquitination. 293T cells were transfected with HA-Ub, treated with either ctrl/CM or 90K/CM for 16 h, and immunoprecipitated with either anti-hemagglutinin (HA) or anti-E-cadherin antibody. Proteasome inhibitor, MG132, was used to block proteasomal degradation of ubiquitinated E-cadherin; (**c**) 90K/CM treatment did not affect E-cadherin mRNA levels. 293T cells were treated with either ctrl/CM or 90K/CM for 16 h, and E-cadherin mRNA level was analyzed by quantitative reverse transcription-polymerase chain reaction (RT-PCR).

In previous work, we showed that 90K downregulates cellular β-catenin levels via ISGylation-mediated proteasomal degradation [19]. The fact that the effects of 90K on E-cadherin were observed at 6 h suggests the involvement of post-translational modification. Therefore, we tested whether the 90K-induced downregulation of E-cadherin was ISGylation-dependent by assessing the conjugation of ISG15 to E-cadherin and whether it was affected by 90K/CM treatment. Our results showed no significant ISGylation of E-cadherin and 90K/CM treatment did not induce any change in the ISGylation of E-cadherin (Figure 4a). However, 90K/CM treatment promoted ubiquitination of E-cadherin (Figure 4b). To rule out the possibility of transcriptional regulation of E-cadherin by 90K/CM treatment, the E-cadherin mRNA level was measured by quantitative reverse transcription-polymerase chain reaction (RT-PCR). Results showed that the mRNA levels of E-cadherin are not affected by 90K/CM treatment in various cells (Figure 4c).

### 2.4. 90K Forms a Complex with E-Cadherin and Decreases the Association between p120-Catenin and E-Cadherin through Affecting Phosphorylation Status of p120-Catenin

**Figure 5:**
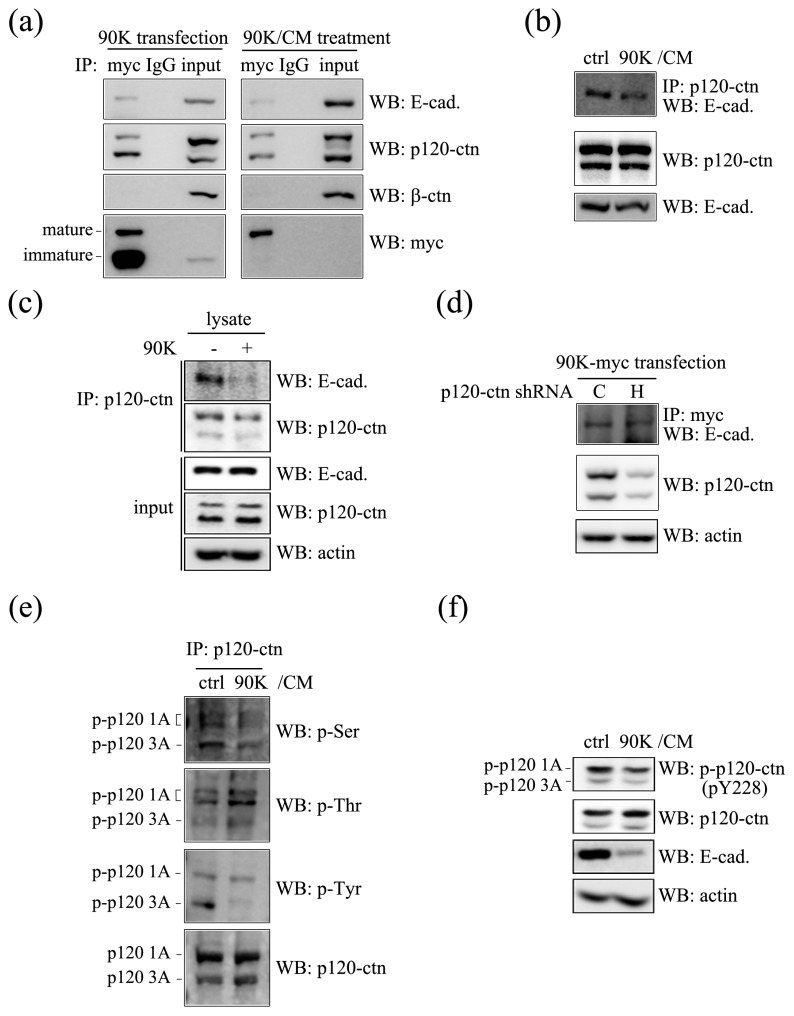
90K interacts with E-cadherin, which affects the phosphorylation status of p120-catenin and thereby decreases the p120-catenin/E-cadherin association. (**a**) 90K was co-immunoprecipitated with E-cadherin and p120-catenin, but not with β-catenin. 293T cells were either transfected or treated with 90K (left panel) or 90K conditioned media (90K/CM) (right panel), followed by immunoprecipitation assay with an anti-myc antibody; (**b**) 90K/CM treatment decreased the interaction between p120-catenin and E-cadherin. 293T cells were treated with either ctrl/CM or 90K/CM for 16 h and immunoprecipitated with an anti-p120-catenin antibody; (**c**) Addition of 90K in 293T cell lysate decreased the interaction between p120-catenin and E-cadherin in vitro. Immunoprecipitation assay was performed by anti-p120-catenin antibody using 293T cell lysates added with or without 90K; (**d**) Knockdown of p120-catenin did not significantly affect the interaction between 90K and E-cadherin. 293T cells were transfected with 90K together with p120-catenin short hairpin RNA (shRNA), which is specific for either canine (C) or human (H) p120-catenin. The shRNA targeting canine p120-catenin, which cannot bind to human p120-catenin, was used as a negative control; (**e**) 90K/CM treatment affected the phosphorylation status of p120-catenin. 293T cells were treated with either ctrl/CM or 90K/CM for 16 h. p120-Catenin immuno-complex beads were followed by western blot analysis using antibodies against phospho-Ser, phospho-Thr, or phospho-Tyr; (**f**) 90K/CM treatment decreased phosphorylation of p120-catenin at Tyr-228 residue. 293T cells were treated with either ctrl/CM or 90K/CM for 16 h, and immunoblotted for phospho-Tyr-228-p120-catenin, p120-catenin, E-cadherin, and actin.

To test whether 90K interacts with E-cadherin, immunoprecipitation assays were performed using HEK293T cells transfected with a 90K-myc expression plasmid. E-cadherin was specifically immunoprecipitated with 90K (Figure 5a, uppermost left panel). p120-Catenin, a well-known component of the adherens junction–E-cadherin complex that interacts with the juxta-membrane domain of E-cadherin and regulates the stability and trafficking of E-cadherin, was also co-immunoprecipitated with 90K, suggesting that 90K can form a trimeric complex with E-cadherin and p120-catenin (Figure 5a, second upper left panel). However, β-catenin, which interacts with the cytoplasmic domain of E-cadherin, was not immunoprecipitated by 90K (Figure 5a, third upper left panel). In transfected cells, ectopically expressed 90K exists in an immature or a mature glycosylated form that is secreted into the medium and acts on neighboring cells in a paracrine manner. To determine whether mature 90K in transfected cells is involved in the trimeric complex, HEK293T cells were treated with 90K/CM, which contains only mature 90K, and lysates were subjected to immunoprecipitation. As shown in the bottom right panel of Figure 5a, 90K-myc was successfully immunoprecipitated from cells treated with 90K/CM. The lack of the mature 90K band in the input lane was likely due to the low amounts present. Consistent with the results obtained using lysates of 90K-transfected 293T cells, both E-cadherin and p120-catenin were specifically immunoprecipitated with 90K, whereas β-catenin was not. These results suggest that mature 90K forms a trimeric complex with E-cadherin and/or p120-catenin (Figure 5a, right panel).

To assess the functional outcome of the 90K/E-cadherin association, we analyzed the effect of 90K on the interaction between E-cadherin and p120-catenin. Considering the reduction in total E-cadherin by 90K, exposure of 90K/CM to subconfluent cells marginally weakened the interaction between p120-catenin and E-cadherin (Figure 5b). However, 90K clearly diminished the interaction between p120-catenin and E-cadherin in vitro (Figure 5c). Unexpectedly, knockdown of p120-catenin did not affect the interaction between 90K and E-cadherin (Figure 5d). These results suggest that 90K interacts with E-cadherin, resulting in the dissociation of the p120-catenin/E-cadherin complex. On the other hand, p120-catenin does not affect the interaction of 90K with E-cadherin.

Changes in the phosphorylation status of p120-catenin affect its binding affinity to various cadherins [29]. As our results showed that 90K/CM treatment weakened the interaction between E-cadherin and p120-catenin, we examined the effect of 90K on p120-catenin phosphorylation. As shown in Figure 5e, 90K significantly decreased p120-catenin phosphorylation at serine and tyrosine residues, but increased it at threonine residues. Particularly, our results showed that phosphorylation of Tyr-228 residue of p120-catenin is decreased by 90K/CM treatment (Figure 5f). These results indicated that 90K forms a complex with E-cadherin, affecting the stability of the adherens junctional complex through inhibiting the interaction between E-cadherin and p120-catenin by altering the phosphorylation status of p120-catenin.

### 2.5. 90K Decreases Membrane Localization of p120-Catenin

**Figure 6:**
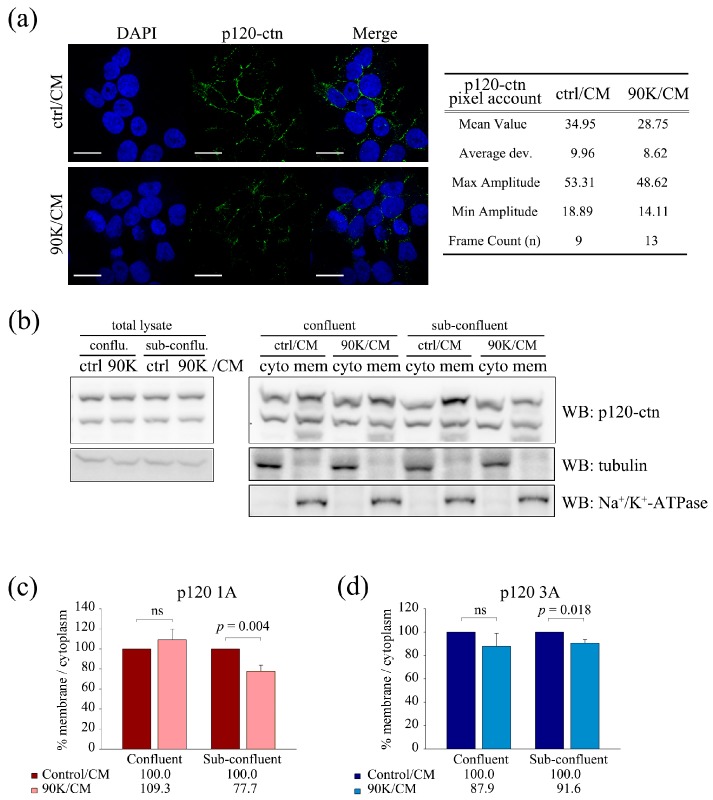
90K affects the cellular localization of p120-catenin. (**a**) 90K conditioned media (90K/CM) treatment decreased the membrane localization of p120-catenin. 293T cells were treated with either ctrl/CM or 90K/CM for 16 h and immunostained using p120-catenin antibody. Representative images are shown in the left panel. Pixel counts for p120-catenin fluorescence are shown in the right panel. Scale bar, 20 μm. (**b**) 90K/CM treatment decreased the membranous fraction of p120-catenin. 239T cells were treated with either ctrl/CM or 90K/CM for 16 h, followed by subcellular fractionation of proteins into either cytoplasmic or membrane fractions. Lysates were then subjected to western blot analysis using an anti-p120-catenin antibody; (**c**,**d**) Quantitative analysis of p120-catenin fractionation. The results of at least seven independent experiments were analyzed to determine statistical significance. ns, no significant difference was found between the indicated groups.

To examine the effect of 90K on the localization of p120-catenin after the dissociation of the E-cadherin–p120-catenin complex, the levels of membrane-bound p120-catenin were measured using two different methods. As shown in Figure 6a, immunostaining experiments showed that the level of membrane p120-catenin was significantly lower in 90K/CM-treated cells than in the untreated controls. Quantitative analysis of p120-catenin fluorescence intensity showed that the mean pixel count was 34.95 (max amplitude: 53.31; min amplitude: 18.89; *n* = 9) in ctrl/CM and 28.75 (max amplitude: 48.62; min amplitude: 14.11; *n* = 13) in 90K/CM-treated cells, indicating an approximately 18% reduction in membrane p120-catenin levels in 90K/CM-treated cells (Figure 6a). To determine the precise fold changes in membrane p120-catenin levels, cells at different confluence levels treated with either ctrl/CM or 90K/CM were separated into membrane and cytoplasmic fractions, and the levels of p120-catenin in each fraction were analyzed (Figure 6b). Quantification of the p120-catenin 1A and 3A isoforms in fractionated lysates showed that the membrane/cytoplasm p120-catenin ratio was decreased in 90K/CM-treated cells, especially in subconfluent cells, and the percent reduction was comparable to that observed in Figure 5a (Figure 6c,d).

### 2.6. p120-Catenin Plays a Pivotal Role in 90K-Induced Downregulation of E-Cadherin and Promotion of Cancer Cell Invasion

**Figure 7:**
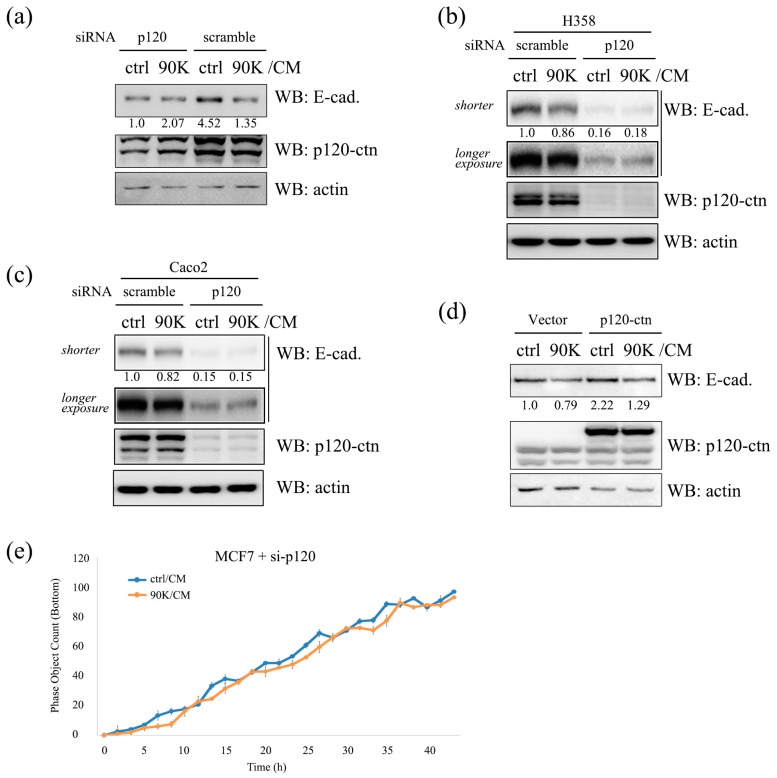
p120-Catenin plays a pivotal role in 90K-induced regulation of E-cadherin level and cell invasion. (**a**–**c**) p120-Catenin-silencing suppressed the downregulation of E-cadherin by 90K/CM. Subconfluent 293T (**a**), H358 (**b**), and Caco2 (**c**) cells transfected with either scramble or p120-catenin small interfering RNA (siRNA) were treated with ctrl/CM or 90K/CM for 16 h and immunoblotted for E-cadherin, p120-catenin, and actin; (**d**) p120-Catenin overexpression did not affect the downregulation of E-cadherin by 90K conditioned media (90K/CM). Subconfluent 293T cells transfected with mock or GFP-p120-catenin 1A were treated with ctrl/CM or 90K/CM for 16 h and immunoblotted for E-cadherin, p120-catenin, and actin. Actin was used as a loading control. Relative values of E-cadherin/actin ratio in triplicate experiments are shown below the E-cadherin panel; (**e**) Invasive potential was not affected by 90K in p120-catenin-silenced cells. Invasion assay was performed with MCF7 cells transfected with p120-catenin siRNA, and subconfluently plated cells were treated with ctrl/CM or 90K/CM. Invaded cells at the bottom of the transwell chamber were monitored using a live-cell imaging device (IncuCyte) with real-time check for the subconfluency of the upper chamber cells, and time-course analyses of the invaded cell number are shown. Values are presented as mean ± SEM. Significant differences between the groups were not found.

To determine whether p120-catenin is required for the 90K-mediated regulation of E-cadherin, silencing and overexpression experiments were performed using a p120-catenin small interfering RNA (siRNA) or p120-catenin expression plasmid. Since 90K treatment primarily decreased the membrane/cytoplasm p120-catenin 1A ratio (Figure 6c,d), a p120-catenin 1A expression plasmid was used. In p120-catenin knockdown cells, E-cadherin levels were decreased (Figure 7a–c, lane 1 vs. lane 3) and the effect of 90K on E-cadherin was not observed (Figure 7a, lane 1 vs. lane 2; Figure 7b,c, lane 3 vs. lane 4). However, in p120-catenin 1A overexpressing cells, E-cadherin was upregulated (Figure 7d, lane 1 vs. lane 3), and the effect of 90K on E-cadherin in subconfluent cells remained intact (Figure 7d, lane 3 vs. lane 4). These results indicated that the downregulation of p120-catenin reduced the ability of 90K to dissociate p120-catenin from the E-cadherin–p120-catenin complex and, consequently, no additional effect was observed, whereas 90K dissociated overexpressed p120-catenin from E-cadherin, thereby decreasing E-cadherin levels in subconfluent cells. Overall, the effects of silencing (Figure 7a–c, lane 1 vs. lane 3) or overexpression of p120 (Figure 7d, lane 1 vs. lane 3) on the E-cadherin level itself emphasizes the strong regulatory effect of p120-catenin on E-cadherin. Indeed, contrary to what was observed in Figure 3e, the invasive potential of MCF7 cells was not affected by 90K/CM treatment if p120-catenin was silenced (Figure 7e). However, it should be noted that DLD1, SW620, A549, and CSC221 cells in which the 90K effect on E-cadherin was diminished (Figure 2d) still did not show a 90K response on E-cadherin in the p120-catenin silencing (Figure S4, lane 3 vs. lane 4). Also, the invasive potential of the cell and phosphorylation of the Tyr-228 residue of p120-catenin were not changed by 90K in these cells (Figure S5). Thus, it is suggested that difference in the p120-catenin level in the cell is not the sole determinant factor for the 90K effect, and another unresolved mechanism may exist for 90K-induced E-cadherin and cell motility regulation.

## 3. Discussion

In the present study, we investigated the effect of 90K on E-cadherin levels. Our main findings were as follows: (1) 90K negatively regulates E-cadherin levels in a cell-population-dependent manner; (2) 90K affects the adhesive and motile properties of subconfluent cancer cells; (3) the 90K modulation of E-cadherin levels is mediated by ubiquitination-dependent proteasomal degradation; (4) 90K interacts with adherens junction proteins, which affects the phosphorylation status of p120-catenin and the association between p120-catenin and E-cadherin; and (5) 90K affects the cellular localization of p120-catenin. However, it should be noted that other factors in the conditioned medium may be responsible to some degree for some of the described effects.

The results of the present study indicate the identification of a new mechanism of destabilizing E-cadherin, especially in low-confluence cancer cells. 90K interacts with p120-catenin at the membrane, modulating p120-catenin phosphorylation and the interaction between p120-catenin and E-cadherin, resulting in changes in the subcellular localization of p120-catenin. Furthermore, 90K interacts with p120-catenin and promotes the dissociation of p120-catenin from E-cadherin. It was reported that the complex between E-cadherin and p120-catenin is critical for the formation and maintenance of the adherens junctions, and disruption of this complex is a hallmark feature of epithelially derived cancers [30]. p120-Catenin, which stabilizes the cadherin–catenin complex, interacts with the E-cadherin juxtamembrane domain (JMD) via masking motifs that are responsible for E-cadherin endocytosis and recycling or ubiquitination-mediated degradation. Moreover, p120-catenin loss or mislocalization can destabilize E-cadherin, and concordant p120-catenin and E-cadherin loss likely favors cancer cell invasion and development of an invasive cancer phenotype [27]. Therefore, in this study, the 90K-induced dissociation of the p120-catenin complex destabilizes E-cadherin.

E-cadherin stability and its interaction with p120-catenin are modulated by p120-catenin phosphorylation mediated by various plasma membrane kinases [29]. In the present study, we showed that 90K decreased p120-catenin phosphorylation at serine and tyrosine residues. Since tyrosine kinases such as Src or receptor tyrosine kinases increase E-cadherin affinity for p120-catenin [31,32], the effect of 90K on the inhibition of tyrosine phosphorylation suggests that 90K destabilizes E-cadherin by promoting its dissociation from p120-catenin. Furthermore, Y228 phosphorylated p120-catenin has a higher affinity for E-cadherin than unphosphorylated p120-catenin in colorectal cancer cells [33]. The effect of phosphorylation of a different serine residue is not clear, because S268 and S269 phosphorylation disrupt the interaction, whereas S873 phosphorylation increases the affinity for E-cadherin [34]. Taken together, these previous findings support our results that 90K promotes p120-catenin dissociation from the E-cadherin complex and alters the phosphorylation status of p120-catenin, thereby destabilizing E-cadherin. However, exactly how mature secreted 90K affects the intracellular p120-catenin phosphorylation process remains elusive.

The downregulation of E-cadherin by 90K was cell-density-dependent, namely, 90K regulated E-cadherin levels in low-confluence cells, whereas its effect was suppressed in confluent cells. Since the formation of stable junction complexes occurs at cell–cell contacts in confluent cells, these results suggest that the regulation of E-cadherin by 90K is junction-complex-status-dependent. Cancer cells show diminished cell–cell adhesion compared with normal epithelial cells [35], and E-cadherin loss or downregulation has been reported in breast, nasopharynx, gastrointestinal tract, pancreas, lung, stomach, kidney, and prostate cancers [36]. Therefore, high serum or tumor levels of 90K promote E-cadherin loss at cancerous epithelial cells, resulting in weak cell–cell adhesion. As the loss of E-cadherin, a well-known inhibitor of invasion, increases the invasive potential of cells, the weakening of E-cadherin-mediated adherens junctions by a microenvironment with high levels of 90K may be a part of a mechanism for cancer metastasis. Indeed, high serum and tumor levels of 90K are associated with metastasis in breast cancer [12,16]. In non-small-cell lung cancer patients, high tissue 90K levels by immunohistochemistry are correlated with distant metastasis, which was detected at a rate of 21% in patients with low 90K expression and 60% in those with high 90K levels [11]. These findings, taken together with the results of the present study, suggest that 90K plays a role in cancer metastasis by promoting the degradation of E-cadherin in low-confluence cells, leading to increased cell motility through the weakening of cell–cell adhesion. When considering that circulating tumor cells in blood vessels likely exhibit low confluence in the cell culture, we hypothesize that 90K may have different effects on cell adhesion and invasion depending on the level of cell density and thereby contribute to association with distant metastasis and poor prognosis in cancer patients with high serum 90K levels. Of potential interest is the cell-density-dependent regulation of β-catenin by 90K. In contrast to E-cadherin, downregulation of β-catenin by 90K was remarkable in confluent cells but not in low-confluence cells, and these may somewhat explain the ambiguous role of 90K on the progression of cancers. That is, 90K exerts antitumor activity through suppressing Wnt/β-catenin signaling by ISGylation-dependent ubiquitination-mediated downregulation of β-catenin via cell surface interaction with CD9/CD82 tetraspanin [19] in a complete cellular microenvironment. However, alternatively, it promotes progression of the disease to malignant by downregulation of E-cadherin after the cell loses their integrity with neighboring cells.

In summary, we here show that E-cadherin associates with 90K and that destabilization of the E-cadherin–p120-catenin interaction by 90K alters p120-catenin subcellular localization and downregulates E-cadherin, resulting in decreased cell–cell adhesion. These effects were seen in low-confluence cells, which will promote escaping cancer cells from primary tumor tissues or blood vessels. Our present data suggest that high 90K levels play an oncogenic role by stimulating metastasis through the induction of E-cadherin degradation, especially in tumor tissues containing weak adherens junctions. Further study is required to completely reveal the roles of 90K in the progression and/or suppression of tumors in various microenvironments.

## 4. Materials and Methods

### 4.1. Plasmids and Antibodies

The generation of the 90K-myc and ISG15-V5 constructs was described previously [19]. HA-Ub was kindly provided by K.Y. Lee (Chonnam National University, Gwangju, Korea). The antibodies used in the present study were as follows: anti-E-cadherin (C20820, BD Biosciences, San Jose, CA, USA; CS#3195, Cell Signaling Technology, Danvers, MA, USA), p120-catenin (610133, BD Biosciences), β-catenin (#9582, Cell Signaling Technology), actin (A2066, Sigma, St. Louis, MO, USA), myc (M047-3, MBL, Woburn, MA, USA), V5 (PM003, MBL), HA (H9658, Sigma), tubulin (sc-5286, Santa Cruz Biotech, Dallas, TX, USA), phospho-serine (P3430, Sigma), phospho-tyrosine (05-321, Upstate, Molsheim, France), phospho-threonine (P3555, Sigma), phospho-Tyr-228–p120-catenin (ab32403, abcam, Cambridge, MA, USA).

### 4.2. Cell Culture, Transfection, and Reagents

HEK293T (human embryonic kidney), CWR22Rv-1 and PC3 (prostate cancer), Caco2, DLD1, and SW620 (colorectal cancer), A549, H358, and H1650 (lung cancer), and MCF7 and BT474 (breast cancer) cell lines were purchased from the American Type Culture Collection (ATCC, Manassas, VA, USA) and the CSC221 (colorectal cancer) cell line was purchased from BioMedicure (San Diego, CA, USA). CWR22Rv-1, PC3, BT474, A549, H358, and H1650 cells were maintained in RPMI1640, and HEK293T, Caco2, DLD1, SW620, MCF7, and CSC221 cells were maintained in DMEM, supplemented with 10% FBS and 1% penicillin/streptomycin at 37 °C in a 5% CO_2_ atmosphere. All cell lines used in the study were authenticated by the ATCC and BioMedicure using short tandem repeat (STR) PCR analysis. For the nonspecific scrambled siRNA (si-scr), we used the All Stars Negative Control siRNA (Qiagen, Hilden, Germany). The siRNA duplexes were prepared and transfected according to a protocol provided with Lipofectamine^TM^ RNAiMAX (Invitrogen, Carlsbad, CA, USA). The short hairpin RNA (shRNA) for p120-catenin was kindly provided by M. Ikura (University of Toronto, Toronto, ON, Canada).

For RT-PCR, the primer set for E-cadherin was as follows: 5′-CGAGAGCTACACGTTCACGG-3′/5′-GGGTGTCGAGGGAAAAATAGG-3′, and that for GAPDH was as follows: 5′-ATCACCATCTTCCAGGAGCGA-3′/5′-AGTTGTCATGGATGACCTTGGC-3′.

### 4.3. 90K Conditioned Media Preparation

The 90K conditioned media (90K/CM) were prepared as previously described [19]. Briefly, HEK293T cells were stably transfected with a 90K-myc expression plasmid and incubated in culture media without serum. After 4 days, the medium was collected and filtered to remove cells and debris. Control conditioned medium was obtained with HEK293T cells transfected with mock vector. The concentration of 90K in 90K/CM (521.5 ng/mL) was determined by enzyme-linked immunosorbent assay (ELISA) [19]. The verified conditioned media were used to treat cells at 10% of the volume of culture media (0.2 mL of conditioned medium was used for 2 mL of culture media in a 6-well plate; ~0.1 µg of 90K/well). Anti-myc antibody was used to deplete functional 90K-myc in 90K/CM.

### 4.4. Immunoblotting and Immunoprecipitation

Immunoprecipitation and immunoblotting were performed as previously described [37]. Immunoprecipitation was performed by incubation of lysates with primary antibody for 16 h at 4 °C, and immunoprecipitates were pulled down using protein G-Sepharose beads (GE healthcare). All results are representative of at least three independent experiments. The intensity of the bands was determined using Multi-Gauge 3.0 densitometry in multiple experiments, and the values of the band were normalized to those of the loading controls. Experimental differences were tested by statistical analysis.

### 4.5. Immunostaining and Image Acquisition

Immunostaining and image acquisition were performed as previously described [38]. Briefly, cells were fixed with 4% paraformaldehyde in PBS, blocked using PBS containing 1% goat serum for 1 h at room temperature, and incubated with primary antibody for 16 h at 4 °C. Cells were then washed in PBS, incubated for 1 h at RT with Alexa Fluor 488-conjugated secondary antibody, and washed in PBS. For confocal analyses, images were visualized and acquired using a TCS SP5 AOBS/Tandem microscope (Leica Microsystems GmbH, Wetzlar, Germany) at Korea Basic Science Institute, Gwangju Center, Korea. Images were analyzed using LAS AF (Version: 2.3.5 build 5379) software (Leica Microsystems GmbH).

### 4.6. Cell Fractionation

Cytoplasmic and membrane proteins were extracted using a Subcellular Protein Fractionation Kit (78840, Thermo Scientific, Waltham, MA, USA). To minimize contamination between fractions, pellets were washed in buffers from the previous step. The % membrane/cytoplasm ratios of p120-catenin 1A and 3A were determined by quantifying each band of membranous or cytoplasmic p120-catenin using the Multi Gauge 3.0 software program (Fujifilm, Tokyo, Japan). All results were determined using at least three independent experiments.

### 4.7. Cell Adhesion Assay

Cell–cell adhesion assays were performed in 48-well plates. MCF-7 cells (2 × 10^5^/well) and Caco2 cells (1 × 10^5^/well) were added to each well in triplicate and incubated for 24 h at 37 °C to prepare the cell-coated plate. While incubating the cell-coated plate, subconfluently plated MCF-7 and Caco2 cells were treated with ctrl/CM or 90K/CM for 16 h. After trypsinization, the cells were labeled with calcein green (Invitrogen #34852, Carlsbad, CA, USA) in PBS for 10 min at 37 °C and then washed three times with PBS. A quantity of 3 × 10^5^ of stained cells were then added to each well in triplicate and incubated for 1 h at 37 °C to allow the cells to adhere to the cell monolayer. After washing, cells remaining attached to the cell monolayer were visualized and counted under a fluorescence microscope.

### 4.8. Live-Cell Analysis of Cell Invasion

For live-cell analysis of cell invasion, the IncuCyte ZOOM Chemotaxis Module and the IncuCyte ClearView 96-well cell migration plate were used. For each insert, 1 × 10^3^ cells in 60 µL medium with 1% FBS were seeded on the Matrigel G (50 µg/mL) coated membrane with 8 µm pores. Fibronectin (20 µg/mL) was added to the lower compartment of the chemotaxis chamber. Images of each insert were taken every 30 min for a period of 40 h. The chemotactic migration from the top to the bottom side of the membrane was quantified as migrated cells on the bottom of the membrane in relation to the total cell number added. The calculation was performed with the IncuCyte ZOOM microscope software 2015A.

### 4.9. Statistical Analysis

All experiments were assayed in multiplicate. Data were expressed as mean ± standard error of the mean. All statistical analyses were performed using the SPSS version 17. Treatment effects were determined using one-way ANOVA post-hoc analysis or Student’s *t* test. A *p*-value < 0.05 was considered significant unless indicated otherwise.

## Supplementary Materials

Supplementary materials can be found at www.mdpi.com/1422-0067/18/12/2601/s1.
