# Supplementary material for: Glycoprotein 90K Promotes E-Cadherin Degradation in a Cell Density-Dependent Manner via Dissociation of E-Cadherin–p120-Catenin Complex
Source: Int J Mol Sci. 2017 Dec 2;18(12):2601. doi: 10.3390/ijms18122601 (PMC5751204; doi:10.3390/ijms18122601)
Supplement: Supplementary file 1 [file ijms-18-02601-s001.zip › suppl. Figures 1,3,4 .pdf]

International Journal of
Molecular Sciences

MDPI

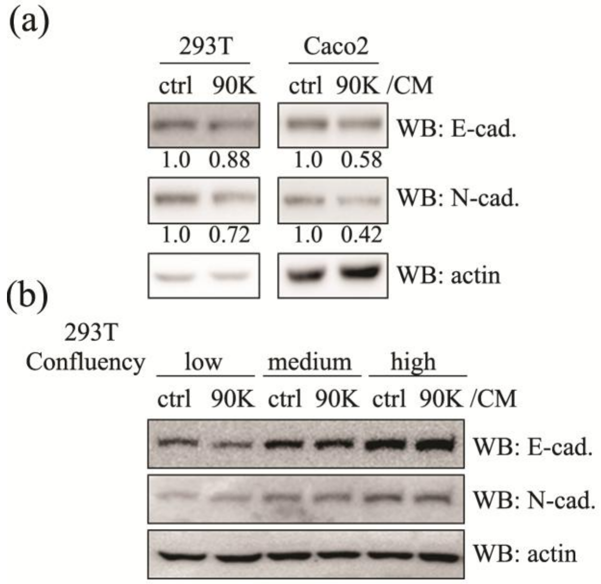

Figure S1. 90K negatively regulates E-cadherin and N-cadherin levels. (a) 90K conditioned media (90K/CM) treatment decreased E-cadherin and N-cadherin levels in 293T cells. Cells were plated to 50% confluence, treated with either control conditioned media (ctrl/CM) or 90K/CM for 16 h, and immunoblotted for E-cadherin, N-cadherin, and actin. (b) The effect of 90K on N-cadherin levels was cell population-dependent. Cells were plated to different confluence levels (high, 70%; medium, 50%; low, 30%), treated with either ctrl/CM or 90K/CM for 16 h, and immunoblotted for E-cadherin, N-cadherin, and actin.

Video clip

Figure S2. 90K increases invasion of the cancer cells. Invasion assay was performed with sub-confluently plated MCF7 and H1650 cells treated with either ctrl/CM or 90K/CM. Invaded cells at the bottom of transwell chamber were monitored using live-cell imaging device (*IncuCyte*).

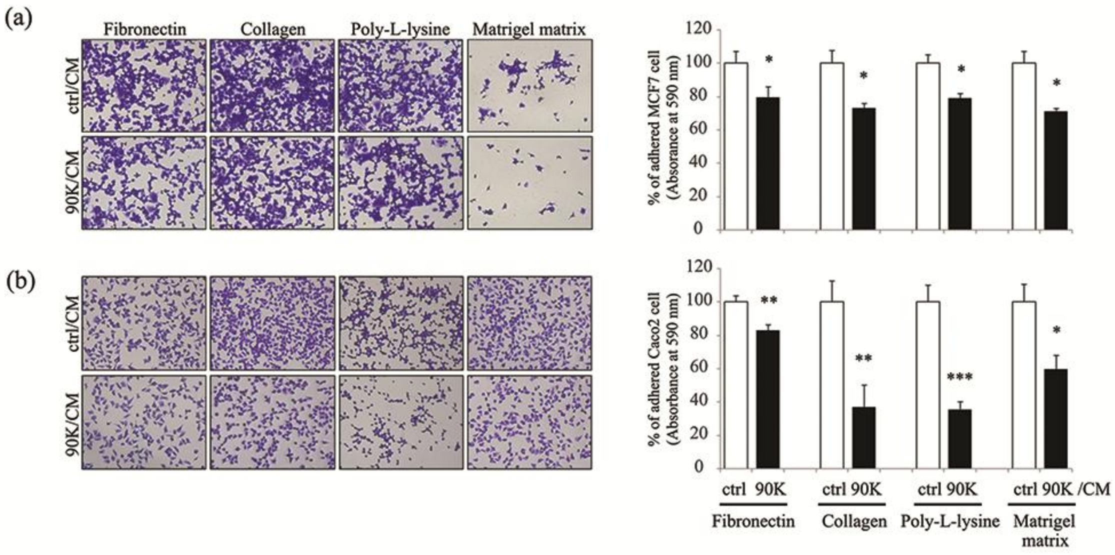

Figure S3. 90K decreases adhesion of sub-confluent cancer cells. (a, b) Cell adhesion was decreased by 90K. Cell adhesion was allowed for 30 min with MCF7 (a) and Caco2 (b) cells to various adhesive substrates. Cells plated on 30% confluence were treated with either ctrl/CM or 90K/CM for 16 h and 3 x 10^5 of cells were added into the adhesive substrate-coated well. Adherent cells were stained and representative images are shown in the left panel and corresponding quantitations of the cell number are shown in in the right panel.

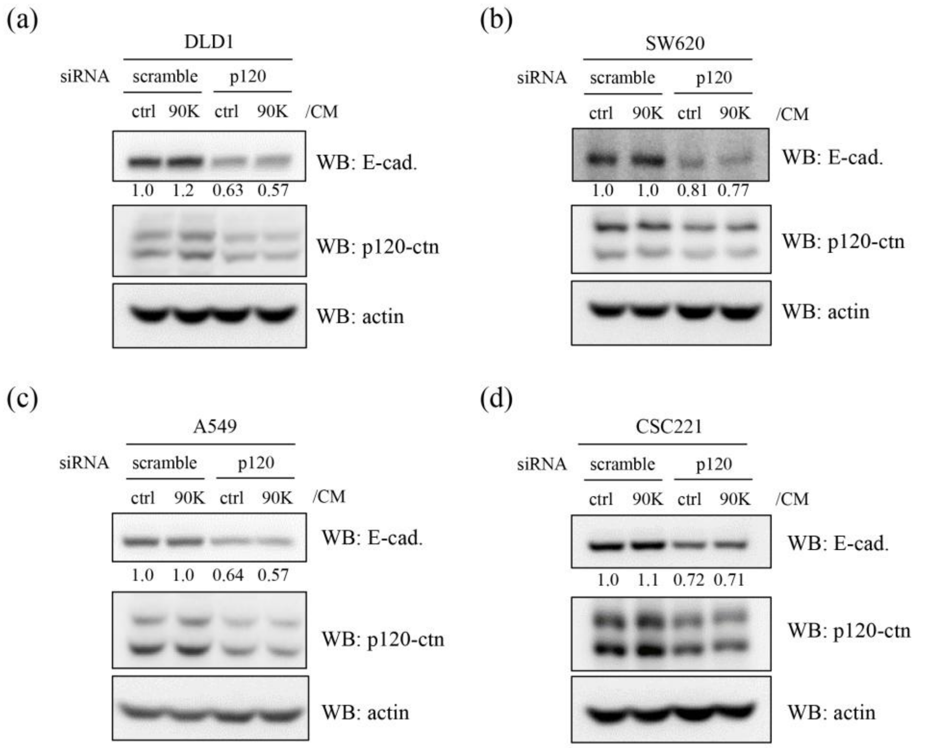

Figure S4. 90K does not affect invasion and phosphorylation status of p120-catenin in DLD1, SW620, A549, and CSC221 cells. (a-e) Invasion assay was performed with sub-confluently plated cells treated with either ctrl/CM or 90K/CM. Invaded cells at the bottom of transwell chamber were monitored using live-cell imaging device (*IncuCyte*) with real-time check for the sub-confluency of the upper chamber cells, and time-course analysis of the invaded cell number are shown. Cell lysates were immunoblotted for p-p120-catenin (Y228), p120-catenin, E-cadherin, and actin (b-e).

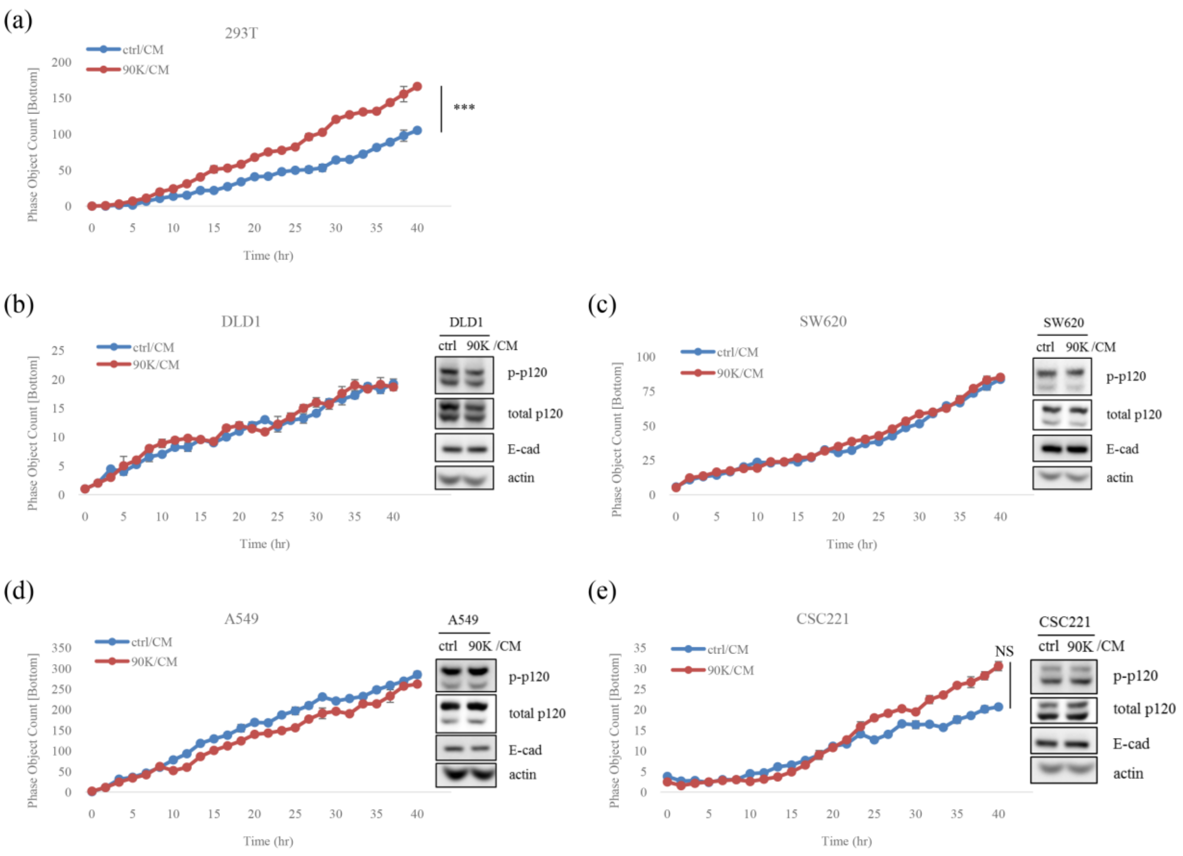

Figure S5. p120-Catenin-silencing does not restore the 90K effect on E-cadherin in DLD1, SW620, A549, and CSC221 cells. (a-d) sub-confluent DLD1 (a), SW620 (b), A549 (c) and CSC221 (d) cells transfected with either scramble or p120-catenin siRNA were treated with ctrl/CM or 90K/CM for 16 h and immunoblotted for E-cadherin, p120-catenin, and actin.
